# d-Serine as a sensor and effector of the kidney

**DOI:** 10.1007/s10157-023-02384-4

**Published:** 2023-07-27

**Authors:** Tomonori Kimura, Shinsuke Sakai, Yoshitaka Isaka

**Affiliations:** 1 Reverse Translational Research Project, National Institutes of Biomedical Innovation, Health and Nutrition (NIBIOHN), Saito-Asagi 7-6-8, Ibaraki, Osaka 5670085 Japan; 2 KAGAMI Project, National Institutes of Biomedical Innovation, Health and Nutrition (NIBIOHN), Saito-Asagi 7-6-8, Ibaraki, Osaka 5670085 Japan; 3 Department of Nephrology, Osaka University Graduate School of Medicine, Yamada-oka 2-2, Suita, Osaka 5650871 Japan

**Keywords:** d-Serine, Glomerular filtration rate, Chronic kidney disease, Kidney transplantation, d-Serine clearance, Diabetes, d-Asparagine, Glomerular filtration rate

## Abstract

d-Serine, a rare enantiomer of serine, is a biomarker of kidney disease and function. The level of d-serine in the human body is precisely regulated through the urinary clearance of the kidney, and its clearance serves as a new measure of glomerular filtration rate with a lower bias than creatinine clearance. d-Serine also has a direct effect on the kidneys and mediates the cellular proliferation of tubular cells via mTOR signaling and induces kidney remodeling as a compensatory reaction to the loss of kidney mass. In living kidney donors, the removal of the kidney results in an increase in blood d-serine level, which in turn accelerates kidney remodeling and augments kidney clearance, thus reducing blood levels of d-serine. This feedback system strictly controls d-serine levels in the body. The function of d-serine as a biomarker and modulator of kidney function will be the basis of precision medicine for kidney diseases.

## Introduction

**Fig. 1:**
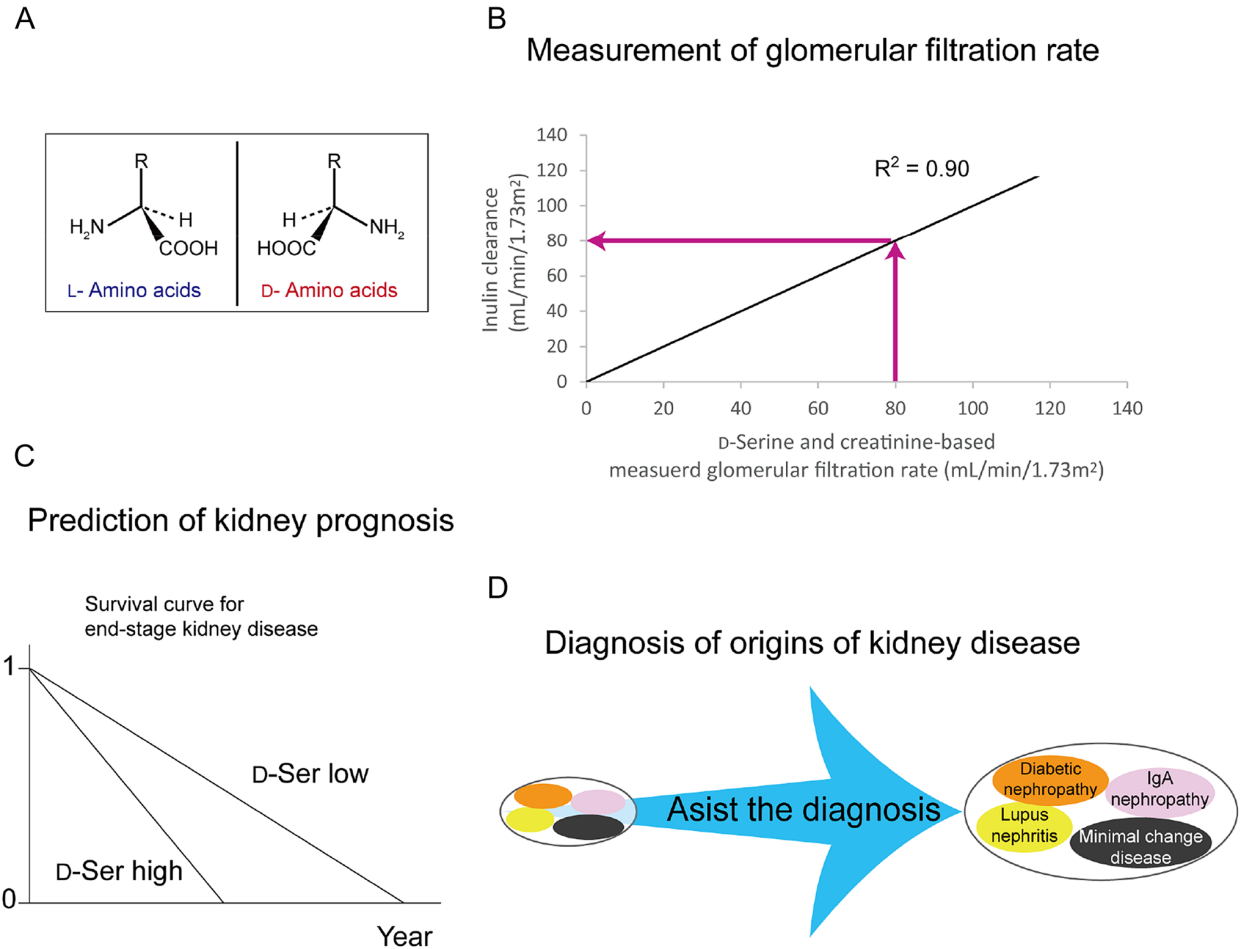
Utility of d-amino acids as biomarkers for past, present and future kidney conditions. **A** Chiral amino acids. l- and d-Amino acids are chiral bodies. They have same molecular weight, bond angle, and bond length, but different characteristics. Only l-amino acids have been regarded to be present in the body until recently. **B** The combinational assessment of d-serine and creatinine clearances achieved a highly precise measurement of glomerular filtration rate, leading to the assessment of present kidney function. **C** The levels of d-amino acids in blood have the capacity to predict the future time to initiate kidney replacement therapy. **D** Several kinds of diseases form a cluster of kidney disease, which are often difficult to distinguish. The blood and urine profiles of d-amino acids vary depending on the origins of kidney diseases, thus assist the diagnosis of origins of kidney diseases, i.e., the past events on the kidney

Chronic kidney disease (CKD) is a global problem, and its prevalence is increasing with the aging of the population. The number of CKD patients is estimated to be 15 million in Japan and 850 million worldwide [1, 2]. The prognosis of CKD patients worsens with the advancement of CKD stages [3], and the medical cost of kidney replacement therapy for patients with end-stage kidney disease is a major socioeconomic problem. Thus, it is critical to prevent CKD from worsening kidney function and progressing to the end-stage. To achieve this, three fundamental problems of CKD should be addressed: (i) insufficient early screening methods, (ii) difficulty in predicting prognosis, and (iii) lack of treatment in the root [4, 5].

d-Amino acids are new biomarkers of the kidney and have the potential to solve the key problems associated with kidney diseases [5]. l - and d-Amino acids are mirror-image enantiomers (Fig. 1A), and d-amino acids were previously regarded as absent in human body. Recent studies have revealed that d-amino acids are present in human body [6–9]. The blood levels of d-amino acids are less than 1–2% of l-amino acids in healthy individuals, whereas these levels increase in patients with kidney disease [9]. Further studies have showed that d-amino acids serve as biomarkers for kidney function and disease (Fig. 1B–D) [5, 9–14]. These findings are attributable to the kidney’s ability to maintain the levels of d-amino acids and a new function of d-amino acids [15, 16].

This review summarizes recent clinical findings and physiological function of d-amino acids. We will describe the scope of CKD problems that d-serine can cover, while proposing a therapeutic strategy based on the unrevealing feedback system for the kidney homeostasis.

## Regulation of d-amino acids dynamics by the kidney

**Fig. 2:**
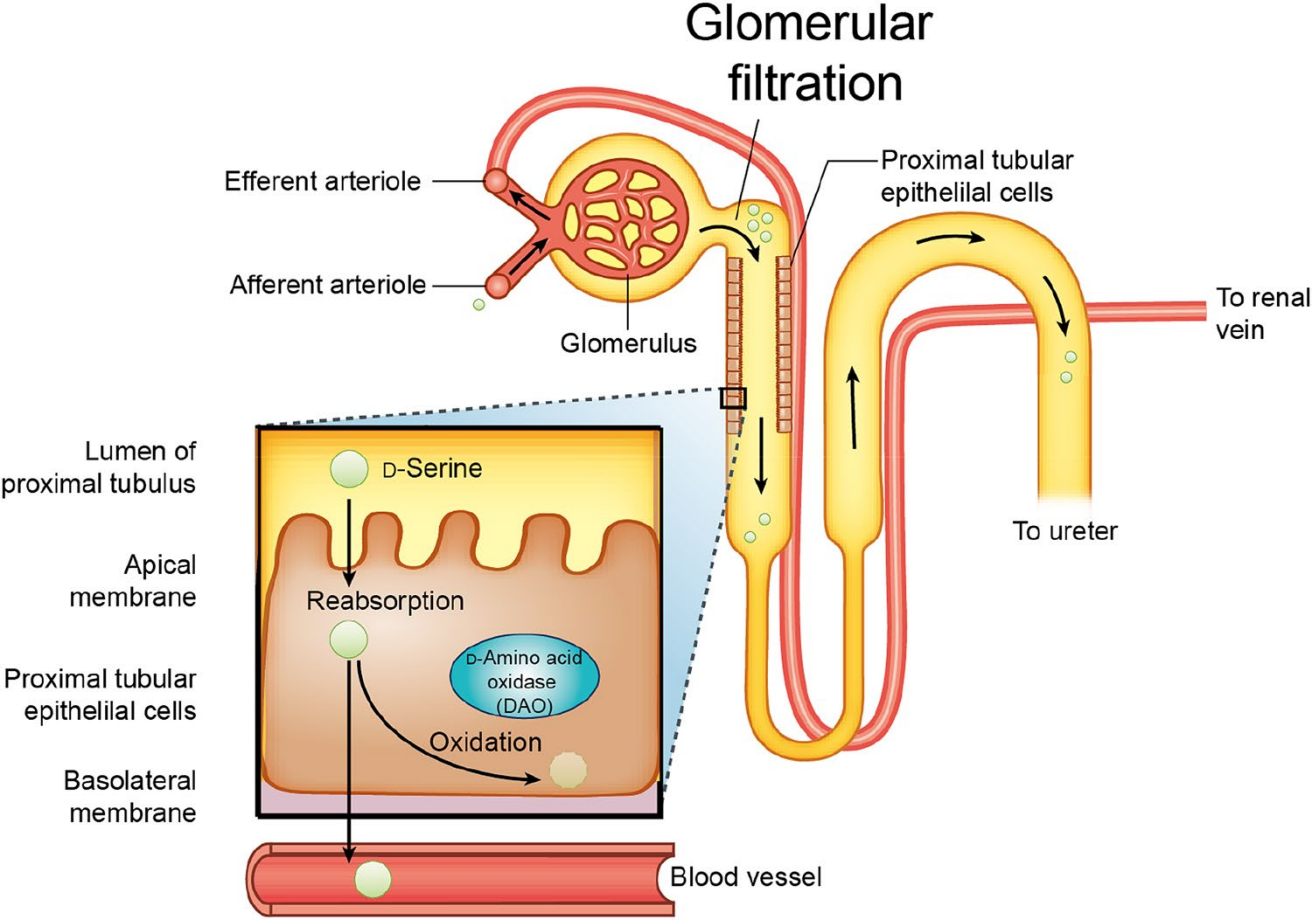
Dynamics of d-amino acids is regulated by the kidney. Blood d-amino acids are delivered to the kidney, where d-amino acids are filtrated in the glomerulus. After filtration, a fraction of d-amino acids is reabsorbed (85% in case of d-serine) in the proximal tubules, some of which are subjected to oxidation by d-amino acid oxidase (DAO). The unabsorbed fraction of d-amino acids is excreted into the urine. In the case of d-serine, about 15% of d-serine is reabsorbed, whereas the rest is excreted in urine

The kidney is a key regulator of the dynamic behaviors of d-amino acids [17]. Among d-amino acids, d-serine is synthesized by an enzyme called serine racemase (SRR). SRR converts l-serine to d-serine in the brain [18, 19], where d-serine functions as a co-agonist of *N*-methyl-d-aspartate (NMDA)-type glutamate receptors that maintain synaptic plasticity [20, 21]. d-Amino acids, including d-serine, are present in food and are relatively rich in fermented food [22]. They are obtained orally from food or are produced by the intestinal microbiota [23]. Subsequently, d-amino acids are absorbed from the intestine into the bloodstream. d-Amino acids in the blood are delivered to each tissue [5].

d-Amino acids in the blood are delivered to the kidney, where they are subjected to glomerular filtration (Fig. 2) [5]. After filtration at the glomerulus, a fraction of d-amino acids is reabsorbed at the proximal tubules [24, 25], whereas the unabsorbed fraction of d-amino acids is excreted into the urine.

The efficacy of reabsorption differs from that of l-amino acids [10]. l-Amino acids have a low urinary fractional excretion ratio (FE), and up to 99% of l-amino acids are reabsorbed at the kidney proximal tubules. On the other hand, the reabsorption efficacies of d-amino acids are highly variable and some of them are very high. The reported median creatinine-based FE for d-serine and d-alanine are 62% and 21%, respectively [10]. Recent study calculated FE using inulin, the standard molecule without reabsorption or secretion, and demonstrated that inulin-based FE for d-serine is 85% [26]. The difference in the FE between chiral amino acids clearly suggests the chiral-selective recognition of amino acids by the kidney.

Within the reabsorbed fraction of d-amino acids, a proportion is oxidized by d-amino acid oxidase (DAO). DAO, which is predominantly present in the kidney proximal tubules [27], oxidizes d-amino acids to generate peroxides [28, 29]. The unoxidized fraction of d-amino acids returns to the bloodstream.

Transporters are the molecular basis for chiral-selective recognition in the kidneys. Like l-amino acids, d-amino acids are actively transported into cells against concentration gradients. To date, *Slc7a10*/*Asc-1* [30], *Slc1a5*/*Asct2*, *Slc5a8*/*Smct1*, and *Slc5a12*/*Smct2* [31] have been reported to play the roles in the delivery of d-serine in the mouse kidney. In the brain, where d-serine is abundant [32], *Slc1a4*/*Asct1*, *Slc1a5*/*Asct2*, and *Slc7a10*/*Asc-1* function as transporters [33, 34]. Transporters for d-amino acids are likely to be common throughout the body. The transporters are differently distributed in the kidney proximal tubules, and cooperate in the reabsorption of d-serine [31].

## d-Amino acids as kidney biomarkers

As the kidney is the key regulator of d-amino acid dynamics, changes in the dynamics of d-amino acids reflect the condition of the kidney. The potential of d-amino acids as kidney biomarkers has rapidly been revealed.

### Measurement of the glomerular filtration rate

Measurement of the glomerular filtration rate (GFR) is the heart of clinical nephrology. GFR describes the flow rate of filtered fluid through the kidney and is calculated as clearance, which is the quantity of a substance in urine that originates from a calculated volume of blood. GFR values of patients are the basis for key clinical decisions, including drug administration design, CKD diagnosis and stage classification, and selection of kidney transplant donors [35, 36]. The clearance of inulin (C-in) is the gold standard for measuring the GFR. Currently, C-in is rarely performed these days since it is time-consuming and labor-intensive—the C-in test requires infusion of exogenous inulin and strictly scheduled samplings of blood and urine [37, 38]. Instead, measurement or estimation of GFR using endogenous kidney biomarkers has been widely adopted. Clearance of creatinine (C-cre) is a precise measure of GFR based on its strong correlation with GFR; however, it has a major proportional bias and overestimates GFR since creatinine is secreted from the kidney tubules into urine [39].d-Serine provides a new measure of GFR, since d-serine is excreted from urine in a GFR-dependent manner [10, 14, 15]. One study analyzed the changes in d-serine clearance in living kidney donors before and after nephrectomy [15]. Nephrectomy induces the hypertrophy of remnant kidney to compensate for the loss of GFR, thus resulting in a 30% reduction in GFR. Correspondingly, d-serine clearance was reduced, and the blood level of d-serine increased after nephrectomy. The blood d-serine level was found to be regulated by d-serine clearance.

**Fig. 3:**
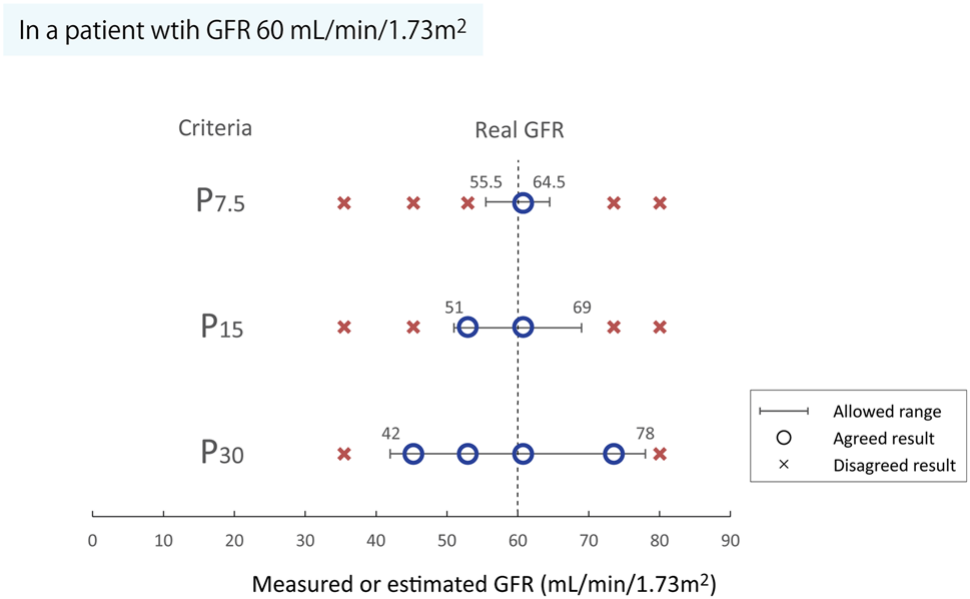
Accuracy to measure and estimate glomerular filtration rate (GFR). For a patient with real GFR of 60 mL/min/1.73m^2^, P_30_, P_15_ and P_7.5_ allow the ranges of 42–78, 51–69, and 55.5–64.5 mL/min/1.73m^2^, respectively, to the GFR monitoring methods. Accuracy can vary dependent on the monitoring methods, therefore, it is necessary to use them based on the purpose, i.e., public health, precision medicine or drug development

Another study demonstrated a close association between d-serine clearance and GFR (Fig. 1B) [14]. In 197 living kidney donors and recipients who underwent C-in test, the levels of d-serine in blood and urine were measured to calculate the clearance of d-serine (C-dSer). C-dSer was significantly and strongly correlated with C-in (*R* = 0.91) similar to C-cre (*R* = 0.93). Importantly, C-dSer was less biased than C-cre as a measure of GFR. Thus, C-dSer can measure GFR precisely with a small bias. Because both C-dSer and C-cre have precision as measures for GFR, the combination of C- dSer and C-cre can measure GFR with the highest precision ever achieved by endogenous molecules (*R* = 0.95), while avoiding biases. Indeed, the equation based on the combination performed well with high ratios of agreement (ratios of 30% and 15% different from C-in [P_30_ and P_15_] of 98.5 [91.4–100] and 89.7 [80.0–95.2], respectively; P_30_ and P_15_ are metrics used to evaluate the agreement of formula as _15_ explained in Box 1; Fig. 3). As a convenient method to measure GFR by endogenous molecules, the combination of C-dSer and C-cre has a high sensitivity for the precise diagnosis and staging of CKD, and helps in formulating drug administration designs or assessing treatment efficacy.

Recently, d-asparagine was also identified as a candidate molecule for an endogenous marker of GFR [26]. In 207 living kidney donors and recipients who underwent C-in test, d-asparagine appeared as an ideal marker for GFR measurement. Like inulin, nearly 100% of d-asparagine is excreted from urine. Therefore, clearance of d-asparagine (C-dAsn) can measure GFR with a negligible level of bias. Both d-serine and d-asparagine have great advantages as GFR markers; d-serine is highly precise, while d-asparagine is less biased. future studies will elucidate how we can utilize these two d-amino acids for measuring GFR.

Box. Accuracy to measure and estimate GFR In studies developing the methods to measure or estimate GFR, P_30_ is often used as metrics of accuracy. P_30_ is the ratio that differs from GFR by less than 30% [40, 41]. In a patient with GFR 60 mL/min/1.73 m^2^, for example, P_30_ allows the range of 42–78 mL/min/1.73 m^2^. If P_15_ is 60%, 60% of patients with GFR 60 mL/min/1.73 m^2^ are correctly assessed as within the range of 51–69, while 40% of patients are below 51 or above 69. From the public health’s point of view, it may be reasonable to assess GFR using the methods developed to optimize P_30_ or P_15_ as long as they are convenient and high in specificity to detect CKD; however, such methods may allow the broader ranges in the assessment of GFR. Resultantly, such methods are weak in the sensitivity for CKD diagnosis and are not suitable for drug dose planning and kidney donor selection. Recent clinical trials of CKD require huge numbers of participants, and resultantly, huge cost [42, 43]. One reason is the variations in the assessment of GFR; they require a large number of participants to draw statistical significance from the highly variable eGFR. Some clinical trials may have ended in false-negative results due to the difficulty in the assessment of GFR. Application of precise methods for GFR assessment can reduce the number of patients needed for the analysis. Development of the methods using more strict criteria, such as P_7.5_, will be the basis of precision medicine in CKD.

### Estimation of GFR

The estimated GFR (eGFR) is a convenient method that is widely used in clinical setting. eGFR is calculated using a combination of age, sex, race and serum levels of creatinine and/or cystatin C. eGFR is relatively less biased [44, 40]; however, its precision is insufficient for the definitive diagnosis and evaluation of the therapeutic effect [41]. Another problem arises in the usage of creatinine. Since it is a waste product from the muscles, the blood level of creatinine is higher in younger people and lower in older people [45]. As a result, kidney function in older people is usually overestimated. For these technical issues, important clinical decisions, such as early diagnosis of CKD, CKD stage classification, and drug administration design, left some uncertainty. The usage of C-dSer can support clinical decisions that require precise and unbiased measures of GFR; however, a better estimation of GFR is still required for the early screening of CKD.

In a cohort consisting of 108 patients with CKD, the levels of d-amino acids, including d-serine, d-alanine, d-proline, and d-asparagine, in the blood correlated strongly with eGFR [9]. The correlations of blood levels of d-amino acids and eGFR were also confirmed in another study of 82 healthy volunteers [46]. To investigate how accurately the blood level of d-serine reflects GFR, blood levels of d-serine were measured in 11 CKD patients and 15 healthy participants who underwent C-in test [10]. The blood level of d-serine was strongly correlated with GFR, which was compatible with those of currently known kidney biomarkers, blood creatinine or cystatin C. The blood d-serine level is useful for estimating GFR, and the addition of the blood d-serine level on conventional kidney markers may improve the estimation of GFR.

### Evaluation of prognosis of CKD

The number of patients with CKD is huge worldwide, and this is another problem that needs to be addressed. Patients with CKD are heterogeneous in their original disease and prognosis. Currently, it is difficult to determine the patients to whom we should focus our medical costs and resources, since we can only estimate the prognosis of patients with CKD insufficiently. Prevention of the worsening of GFR is key to reducing the number of end-stage kidney disease patients requiring kidney replacement therapy and the risk of lethal cardiovascular events, the latter of which increases with the worsening of GFR [3].

d-Amino acids, including d-serine, are useful for evaluating CKD prognosis (Fig. 1C). In a longitudinal cohort of 108 CKD patients refereeing nephrologists, higher levels of d-amino acids, such as d-serine, d-asparagine, d-proline, and d-alanine in the blood were associated with worse prognosis: earlier initiation of kidney replacement therapy or death [9]. In patients with higher blood levels of d-amino acids, the hazard ratios adjusted for clinical factors ranged from 2 to 4, depending on the type of d-amino acid.

### Diagnosis of origin of kidney disease

CKD is heterogeneous in its origin, including immunological, diabetic, aging-related, rare, intractable, and unknown causes. Pathological findings from kidney biopsy are used to estimate the prognosis and to determine the treatment; however, kidney biopsy is risky and is usually performed only once when its merit surpasses the risk [47, 48]. A major concern is the diagnosis of diabetic nephropathy (DN). DN, one of three major complications of diabetes mellitus, has recently become the primary cause of kidney replacement therapy [49, 50].

Clinically, it is often difficult to distinguish DN from chronic glomerulonephritis complicated by diabetes. Proteinuria is a risk factor for prognosis, but the range of proteinuria is wide in DN [51], making it difficult to differentiate other diseases, including minimal change disease [52]. Kidney biopsy is often avoided in patients with diabetes because of complications of cardiovascular diseases and the use of anticoagulant drugs. Therefore, diabetes patients with proteinuria are clinically diagnosed to have diabetic kidney disease (DKD) [53]. Currently, biomarkers that can help diagnose the origin of kidney diseases are insufficient.

Unlike the blood level of d-serine, the urinary excretion of d-serine in patients with CKD is not related to GFR; instead, it is affected by the presence of kidney diseases [10]. Accordingly, the simultaneous assessment of blood levels and urinary excretion of d-serine, that is, intra-body dynamics of d-serine, is useful for monitoring kidney function and disease activity (Fig. 1D) [17].

d-Serine levels in the blood and urine were measured in patients with six types of biopsy-proven kidney diseases [12, 13]. The profiles of d-serine varied depending on the origin of the CKD. These include lupus nephritis, a complication of systemic lupus nephritis (SLE), DN, and minimal change disease. Patients with DN have a unique plasma-high FE-high profile. This profile is useful in distinguishing DN from other kidney diseases that cause proteinuria. The profile of d-serine varies depending on the origin of kidney disease, and may assist in the diagnosis of kidney disease.

Another study shows that d-alanine also has the capacity to discriminate diabetic patients in a group of patients with CKD [54]. Measurements of the combinations of d-amino acids may assist the better diagnosis of the origin of kidney diseases.

### Monitoring of recovery of kidney function

The d-serine profile has a potential to assess the recovery of kidney function. In a patient with a severely and acutely injured kidney due to SLE, the blood level of d-serine was extremely high because of the loss of kidney function [11]. During the recovery course, blood d-serine levels quickly declined in parallel with the serum creatinine levels. The urinary excretion of d-serine, on the other hand, transiently increased, followed by normalization of blood d-serine level. These dynamic d-serine profile can be used to monitor disease activity and treatment effects.

In line with this, the blood levels of d-serine and d-alanine were also correlated with eGFR in patients with acute kidney injury [55, 56]. The higher blood levels of d-amino acids reflect decreased kidney function even under acute phase of kidney injury.

## Function of d-serine in kidney

### Toxicity at high doses and safety at low doses of d-serine in kidney

The kidney regulates the intra-body dynamics of d-serine by urinary clearance. In other words, the kidney is continuously exposed to d-serine delivered from renal artery, which is comprised of 20% of cardiac output. When administered intravenously, d-serine is predominantly delivered to the kidney [15]. Then, what is the function of d-serine in the kidney? Classical studies have demonstrated that treatment with excessive amounts of d-serine is toxic and causes acute tubular necrosis within a day [57]. However, the mechanism of d-serine toxicity remains unclear. One candidate for this is peroxide, which is generated through the oxidation of d-serine by DAO; however, this mechanism could not explain why d-alanine, another substrate for DAO to generate peroxide, is not toxic [58]. In vitro analysis showed that treatment of d-serine at high dose (20 mM) was toxic to human kidney tubular cell lines, induced inflammatory response, and suppressed cellular proliferation [59]. Higher doses of d-serine likely induce the direct toxicity against kidney tubular cells.

In contrast, studies, including human clinical trials, using d-serine at lower doses were reported to be safe [60]. Above all, the presence of a toxic dose of d-serine suggests a physiological role at the lower physiological level. Only 1–2% out of approximately 100 μM of serine is d-serine, and the maximum value in CKD patients can reach up to 17 μM [9, 10]. Now that the level of d-serine in humans is much lower than those tested in classical studies, the function of d-serine at the physiological level needs to be investigated.

### Cellular proliferative effect of d-serine in kidney

The blood level of d-serine increases in living kidney donors after nephrectomy because of its reduced clearance, whereas nephrectomy induces enlargement of the remnant kidney [15]. To investigate the physiological effects of d-serine, mouse models of unilateral nephrectomy were treated with a low dose of d-serine [15]. Treatment with d-serine promoted proliferation of the proximal tubules, where d-serine is reabsorbed. Consequently, d-serine treatment accelerated kidney remodeling. The cellular response has chiral specificity, and the cellular proliferative effect of d-serine is superior to that of l-serine in kidney-derived cells at the physiological level.

mTOR, a sensor of l-amino acids, is known to play a role in remnant kidney enlargement [15]. In vitro analysis showed that the addition of a low dose of d-serine to l-amino acids augmented signals from mTOR to mediate cellular proliferation [15]. The ability of d-serine to enhance mTOR signaling may provide a new therapeutic approach for the chronically damaged kidney.

### Protective effect of d-serine and d-alanine in acute kidney injury

The blood levels of d-amino acids also increase in mouse AKI models and in human AKI patients [30, 55, 56, 61], and some studies examined the significance of d-amino acids under these conditions. Treatment with d-serine ameliorates kidney injury in ischemia/reperfusion (I/R) injury mice and reduces hypoxic cellular toxicity in kidney cell lines [55]. d-Alanine was also reported to have a protective effect in mouse form I/R injury AKI model [56]. Associations between I/R injury and abnormal d-serine metabolism by the gut microbiota have also been suggested [55], and d-serine may be beneficial against AKI either directly or by altering the gut microbiota.

### Immunological and anti-viral function

The pathology of kidney diseases involves immunological processes, and d-amino acids play an immunological role. In high-IgA (HIGA) mice, a model of IgA nephropathy, the activity of DAO is decreased because of missense mutations in the *Dao* gene [16]. Accordingly, the blood d-alanine levels were higher in HIGA mice than in control mice. d-Amino acids promote the survival of B cells, and loss of d-amino acid catabolism results in excessive IgA production. Goblet cells in the intestine secrete d-amino acid oxidase (DAO), which oxidizes d-amino acids produced by microbiota [23]. The product of this reaction is peroxide, which suppresses the growth of pathogenic bacteria. d-Amino acids may link the pathology of IgA nephropathy and microbiota.

d-Amino acids also have a protective role against viral infections. Treatment of d-alanine improved the survival and prognosis of mice infected with either Influenza A virus or SARS-CoV-2 [62]. d-Alanine also reduced viral titers in lungs of mice infected with Influenza A virus [62]. Although the mechanism remains elusive, d-alanine likely plays homeostatic role in body. Upon severe viral infections, blood levels of d-amino acids decrease to the levels lower than the normal ranges [63], and supplementation of d-alanine to maintain its blood level may be effective. Impaired kidney function is a risk for the worsening of viral infectious diseases, including COVID-19 [64]. The abnormal metabolism and dynamics of d-amino acids, which are the key features of the kidney diseases [55, 56], may be associated with worse prognosis of viral infectious diseases in patients with kidney diseases.

### Therapeutic potential of d-amino acids

The basis of CKD treatment is to prevent the worsening of kidney function. Current therapeutic options for kidney diseases are largely supportive. d-Amino acids play protective roles in chronically or acutely damaged kidney [15, 55, 56, 65], and the cellular proliferative function of d-amino acids may provide an opportunity to recover reduced kidney function [15]. In the absence of treatment in roots, it could be a great achievement if d-amino acids recover kidney function, even in a limited fraction. The immunomodulatory function of d-amino acids is also a potential target for resolving the origin of kidney diseases [16]. These directions, which may lead to the development of future therapeutics, have scarcely been investigated thus far.

## Chiral feedback system of d-serine

**Fig. 4:**
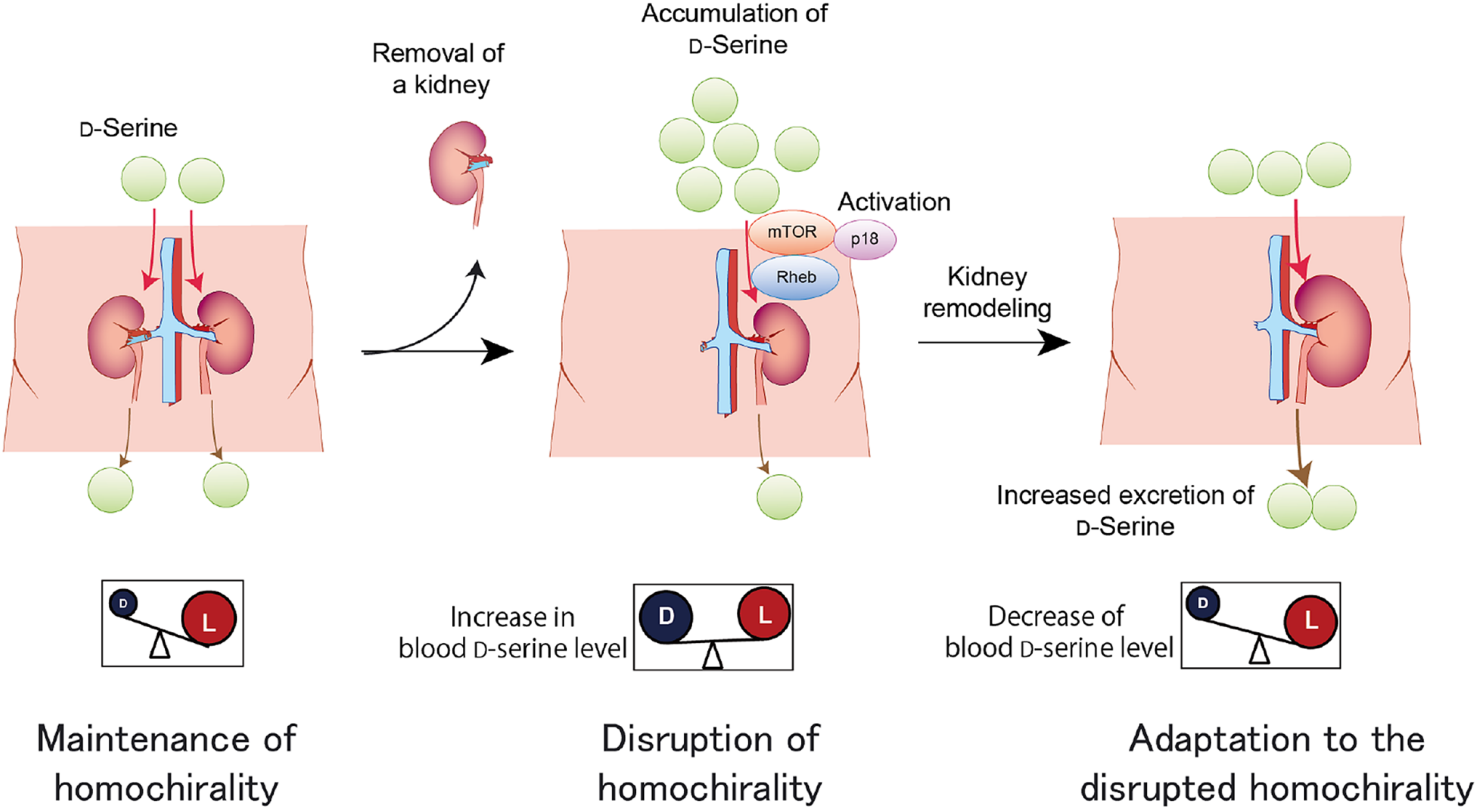
Chiral feedback system. d-Serine in blood is continuously excreted into urine and its level is strictly regulated by the kidney (maintenance of homochirality). Once kidney function is reduced, urinary excretion of d-serine is reduced and the blood level of d-serine increases (disruption of homochirality). The increased blood d-serine, in turn, promotes compensatory kidney remodeling through mTOR signals and an increase in the glomerular filtration rate (GFR) of the remnant kidney. Accordingly, the initial increase in blood d-serine levels is reduced to match the GFR of remodeled kidney (adaptation to the disrupted homochirality). Strict regulation of chirality is a key feature of life

The dual functions of d-serine as a biomarker of kidney and as effector in the kidney are emerging. These functions form a feedback system for d-serine dynamics (Fig. 4).

d-Serine in the bloodstream is continuously excreted from the urine by the kidneys. This regulation is strict enough to reflect GFR. Once kidney function and urinary excretion of d-serine reduced, the blood d-serine level increases. At its elevated level, d-serine promoted the remnant kidney enlargement in the nephrectomy model mice [15]. Therefore, the increased blood d-serine level, in turn, promotes compensatory kidney remodeling and increases the GFR of the remnant kidney and urinary excretion of d-serine. Accordingly, the initial increase in blood d-serine levels is reduced to match the GFR of the remodeled kidney. The blood d-serine level is regulated by a feedback system, where the kidney plays a central role. This feedback system forms the basis for the tight regulation of blood d-serine level by the kidney. Lack of response to blood d-serine levels may indicate the presence of kidney disease. This may also be a feature of the senescent kidneys. The cellular proliferative effect of d-serine is weak in senescent cells [15]. Senescent kidneys may shrink with age because they cannot respond to d-serine stimuli.

One excellent part of this system is that it relies on GFR marker, d-serine. Sensing the level of d-serine as a marker of GFR, kidney remodels its size autonomously. Another benefit of this system may be the chiral regulation. Tight control of chirality is an essential feature of living things on Earth [66], while dysregulation of chirality is associated with diseases [9, 32]. By sensing the chiral disturbance, the kidney modifies its function to control chirality.

## Detection of d-amino acids

Since its presence was suggested more than 80 years ago [28], the role of d-amino acids in the kidney was undetermined until recently. The main reason for this was the difficulty in their measurement. Because of their trace nature, the measurement of d-amino acids is easily affected by abundant l-amino acids and a wide variety of intrinsic compounds present in human samples [5, 67]. While a variety of methods have been introduced to measure d-amino acids [7, 8, 66–70], it is important to select the methods suitable for its purpose. When d-amino acids are used as biomarkers, a subtle difference in the measured results greatly affects the clinical decision; thus, their levels need to be measured precisely [5].

Recently, a two-dimensional high-performance liquid chromatography (2D-HPLC) system was introduced for the precise measurement of d-amino acids [67, 71]. This 2D-HPLC system utilizes two types of columns, one for separating each amino acid and another for chiral separation. The measured blood concentration of d-serine by 2D-HPLC can be applied to measure GFR [14], confirming the precision of the measurement.

## Conclusions

d-amino acids are biomarkers for kidney function and disease activity. Among the d-amino acids, d-serine can provide comprehensive information for the management of kidney diseases. eGFR provides a method to screen for CKD as it was originally introduced from the public health perspective, whereas measurement of d-serine has achieved the precise measurement of GFR, which is of great value in clinics. Measurement of d-serine is useful in situations such as clinical decision-making and drug development, where precise measurement of GFR is necessary. In addition to the GFR (current), d-serine also provides information on the origins of kidney diseases (past) and prognosis of kidney diseases (future). By providing past, present, and future information simultaneously, d-serine will greatly help clinicians to better care for patients with CKD.

In addition to its function as a biomarker, d-serine promotes cellular proliferation for kidney remodeling. d-serine mediates proliferation of proximal tubular cells [15], where d-serine is reabsorbed [24, 25]. This function is part of the chiral feedback system that strictly controls blood d-serine levels. Since the clearance of d-serine closely reflects GFR, the kidney may use d-serine as an indicator to determine its response, that is, increasing the size when the blood d-serine level is high, while maintaining the size when it is low. d-serine is a sensor of kidney condition and is an effector of the kidney. Through the control of kidney function, d-serine feedback system contributes to homeostasis.

In the future, applying d-serine clearance could reduce the burden of drug development for kidney diseases. Currently, eGFR is often used as a readout to estimate the improvement or maintenance of GFR following drug administration. In clinical trials, the imprecision of GFR estimation can easily lead to a beta error, that is, false-negative results. To avoid this, clinical trials aimed at treating CKD have utilized a large number of patients. d-serine-based GFR is preferable in terms of precision in the measurement of GFR and can greatly reduce the number of trials required. Clarifying the molecular basis of chiral feedback systems is a promising path for drug discovery for kidney diseases from a new perspective. In addition to d-serine, the roles of other d-amino acids remain to be elucidated. From these studies, novel biomarkers and physiological functions of d-amino acids will be identified. d-Amino acids improve the clinical quality from a new perspective.
